# Comparing Chinese-language large language models for caregiver questions about developmental dysplasia of the hip

**DOI:** 10.3389/fped.2026.1919701

**Published:** 2026-09-03

**Authors:** Fangyuan Wang, Xianhong Li, Yinghui Guan, Jiaqi Tian, Le Xu, Bing Huang, Jianhui Xie

**Affiliations:** 1 Xiangya School of Nursing, Central South University, Changsha, China; 2 Department of Orthopedics, The Affiliated Children's Hospital of Xiangya School of Medicine, Central South University (Hunan Children's Hospital), Changsha, China; 3 Hospital Administration Office, The Affiliated Children's Hospital of Xiangya School of Medicine, Central South University (Hunan Children's Hospital), Changsha, China

**Keywords:** caregiver education, ChatGPT-4o, Chinese-language health information, DeepSeek-R1, developmental dysplasia of the hip, guideline concordance, large language models, patient education

## Abstract

**Background:**

Large language models (LLMs) are increasingly used for caregiver-facing health information, but their reliability in Chinese-language pediatric orthopaedics remains uncertain. This study evaluated whether responses to developmental dysplasia of the hip (DDH) questions were clinically accurate, aligned with Chinese guidance, and educationally usable.

**Methods:**

We compared ChatGPT-4o and DeepSeek-R1 using 53 Chinese-language DDH questions, including 31 caregiver-oriented frequently asked questions and 22 guideline-derived items. Each model generated one response per question using a standardized single-turn, five-sentence prompt. Six blinded pediatric orthopaedic surgeons rated clinical accuracy and guideline concordance. Paired model comparisons, inter-rater reliability, and exploratory formula-based readability were assessed.

**Results:**

DeepSeek-R1 had higher clinical accuracy than ChatGPT-4o across 31 caregiver-oriented questions (mean question-level score 4.70 [SD 0.15] vs. 3.69 [0.28]; *P* < 0.001) and higher guideline concordance across 22 items (4.44 [0.22] vs. 3.82 [0.33]; *P* < 0.001). Average-rating reliability was good for clinical accuracy (ICC(2,6) = 0.826, 95% bootstrap CI 0.770−0.863) and moderate for guideline concordance (ICC(2,6) = 0.519, 95% CI 0.377−0.621). Formula-based readability findings were mixed: unadjusted tests indicated lower FKGL, FOG, and SMOG values for ChatGPT-4o, but only FOG and SMOG remained statistically significant after Holm adjustment. Surgery-related observations were exploratory and descriptive.

**Conclusions:**

Under the tested single-query and five-sentence conditions, DeepSeek-R1 achieved higher expert-rated clinical accuracy and Chinese-guideline concordance. The findings describe specific model-platform configurations rather than a permanent model ranking. Professionally reviewed LLM responses may help clinicians reinforce routine DDH education, but individualized diagnostic and treatment guidance, especially for surgery-related questions, should remain clinician-led. Caregivers should use chatbot information only as a supplementary resource.

## 1 Introduction

Large language models (LLMs) are increasingly used as accessible sources of health information by patients, caregivers, healthcare learners, and clinicians. Their conversational interfaces allow users to ask open-ended questions and receive immediate explanations, which makes them attractive for patient-facing education and health information support. At the same time, studies in healthcare have repeatedly raised concerns about factual accuracy, response consistency, missing context, unsupported references, and the potential for clinically harmful advice when LLMs are used as primary information sources (1–4). These concerns are particularly relevant when the user is not a health professional and may have difficulty judging whether a response is complete, current, or locally applicable.

Developmental dysplasia of the hip (DDH) is a common pediatric orthopaedic condition that requires repeated communication between clinicians and families across the care pathway. Caregivers often seek information about early signs, screening, imaging, bracing, indications for surgery, postoperative rehabilitation, follow-up, and long-term prognosis (5–7). The information needs of these caregivers are not limited to simple definitions. Many DDH-related questions require explanation of age-specific management pathways, imaging thresholds, uncertainty in treatment decisions, and reasons why individualized clinical assessment is necessary. Inaccurate or oversimplified information may therefore increase anxiety, create unrealistic expectations, or delay appropriate consultation.

LLM-based tools have recently been evaluated in several medical information and patient education settings, including patient education tools, guideline-informed conversational systems, and sources of healthcare-related decision information (4, 8, 9). These studies emphasize that LLM evaluation should move beyond simple answer correctness and should also consider clarity, usefulness, safety, guideline alignment, and the distinction between general education and clinical decision support. Similar considerations are important in DDH, where LLM-generated responses may be useful for reinforcing routine education but may be unsafe if used to make surgical or diagnostic decisions without clinician oversight.

Chinese-language DDH information poses additional challenges. ChatGPT-4o is widely used as an international reference model in medical AI research, whereas DeepSeek-R1 has become a highly accessible Chinese-oriented LLM (10–12). However, model performance may vary by language, medical context, local terminology, and the clinical recommendations used as benchmarks. For DDH, the 2023 Chinese DDH guideline provides an important local reference standard for evaluating whether generated information is not only plausible but also aligned with recommendation-based clinical content (13). Existing DDH-related LLM studies have more often focused on general model performance or international guideline scenarios (14–16), leaving limited evidence on Chinese-language caregiver education and Chinese guideline concordance.

Therefore, this study evaluated ChatGPT-4o and DeepSeek-R1 for Chinese-language DDH information support using a caregiver-oriented and guideline-informed question set. The primary objectives were to compare the models in terms of clinical accuracy and guideline concordance with the 2023 Chinese DDH guideline and to explore formula-based readability and safety-related limitations relevant to caregiver-facing education. Expert observations concerning comprehensibility were treated as descriptive only because complete item-level comprehensibility data were unavailable; comprehensibility was not considered interchangeable with formula-based readability.

## 2 Methods

### 2.1 Study design

**Figure 1:**
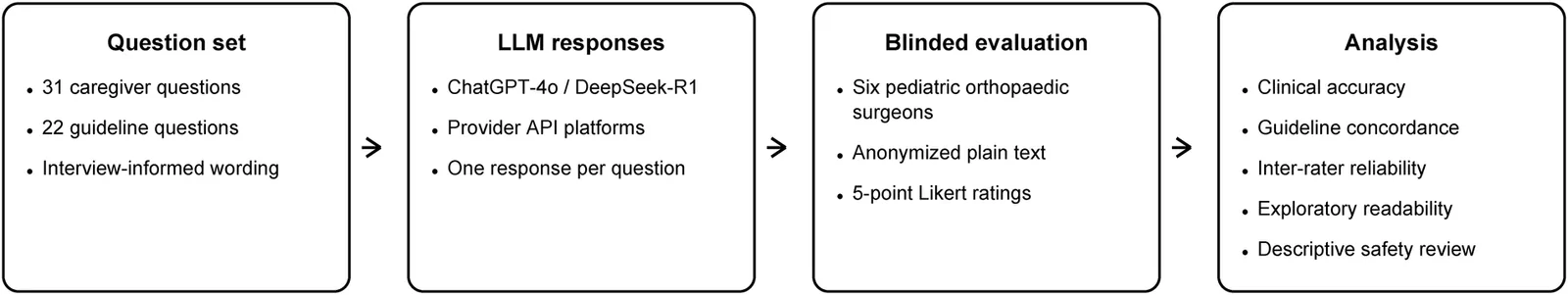
Study workflow for evaluating Chinese-language large language model responses to caregiver questions about developmental dysplasia of the hip. The evaluation set comprised 31 caregiver-oriented questions and 22 items derived from the 2023 Chinese guideline for developmental dysplasia of the hip. ChatGPT-4o and DeepSeek-R1 each generated one Chinese-language response per question through their respective provider application programming interface platforms. Six blinded pediatric orthopaedic surgeons evaluated the responses using 5-point Likert scales. The analyses included clinical accuracy, guideline concordance, inter-rater reliability, exploratory formula-based readability, and descriptive safety review.

This study was designed as a cross-sectional comparative evaluation of Chinese-language LLM-generated responses to caregiver-oriented questions about DDH. The study included two stages: development of an interview-informed and guideline-informed DDH question set, followed by blinded expert evaluation of model-generated responses (Figure 1).

Interview material was used to improve the relevance and wording of caregiver-oriented questions, while guideline-derived questions were used to evaluate alignment with local recommendation-based content. The primary analysis was quantitative and based on blinded expert ratings. The study therefore evaluated the quality, guideline alignment, educational usability, and safety of LLM-generated information rather than clinical diagnosis, treatment efficacy, or patient outcomes.

### 2.2 Development of the DDH caregiver question set

The question set was designed to reflect both real-world caregiver information needs and recommendation-based clinical content. First, an initial pool of candidate questions was generated through a structured review of five curated online medical information sources. These candidate questions were refined using semi-structured interview material from 10 children with DDH and their primary caregivers who had provided consent for the use of interview data. The interview-informed refinement focused on recurring caregiver concerns related to symptom recognition, diagnosis, bracing, treatment decision-making, rehabilitation, follow-up, and long-term prognosis.

Overlapping or ambiguous questions were removed, and wording was revised to reflect the language that caregivers would be likely to use when seeking information. This process resulted in 31 caregiver-oriented high-frequency clinical questions across the DDH care pathway. Because the interview material was used for question development rather than for a standalone qualitative thematic analysis, detailed qualitative themes are not reported separately.

Second, to evaluate alignment with locally relevant recommendation-based content, a guideline-informed question set was developed with reference to the Chinese guideline for the diagnosis and treatment of developmental dysplasia of the hip in China (2023 edition). Key diagnostic and treatment recommendations were converted into 22 structured question items, including 6 diagnostic and 16 treatment-related questions. Together, the caregiver-oriented frequently asked question (FAQ) set and the guideline-informed set comprised 53 Chinese-language DDH questions. All questions and model-generated responses are provided in the Supplementary Materials. The characteristics of the final DDH question set are summarized in Table 1.

**Table 1:** Composition, sources, and content domains of the 53-question Chinese-language evaluation set for developmental dysplasia of the hip.

Question category	Main source	No. of questions	Content focus
Symptom recognition and diagnosis	Caregiver-informed high-frequency questions	9	Early signs, screening, imaging, diagnostic explanation
Treatment and decision-making	Caregiver-informed high-frequency questions	10	Bracing, treatment timing, management options, decision-related questions
Rehabilitation and follow-up	Caregiver-informed high-frequency questions	7	Follow-up schedule, home care, recovery, rehabilitation, and annual review questions
Prognosis and long-term management	Caregiver-informed high-frequency questions	5	Long-term prognosis, recurrence concerns, growth, function, and heredity
Diagnostic guideline items	Chinese DDH guideline (2023 edition)	6	Etiology, risk factors, clinical manifestations, imaging evaluation, differential diagnosis, classification
Treatment guideline items	Chinese DDH guideline (2023 edition)	16	Assessment, Pavlik harness management, reduction, osteotomy, adolescent/adult management, total hip arthroplasty-related recommendations

DDH, developmental dysplasia of the hip; FAQ, frequently asked question.

### 2.3 LLM selection and response generation

Two LLMs were selected for comparison: ChatGPT-4o (OpenAI) and DeepSeek-R1 (DeepSeek AI). Responses were generated on 10 July 2025 through the models' respective provider API platforms; the models were not accessed through a shared third-party interface. The retained study records identified the models by product name but did not include exact snapshot identifiers. Generation parameters, including temperature, top_p, and maximum output length, were not manually tuned or harmonized, and each model retained the provider-specific default settings available at the time of testing. The retained records confirm online API access but do not document whether external browsing, search, or retrieval tools were invoked. Retrieval capability, search scope, and language coverage therefore could not be compared. Accordingly, this study should be interpreted as a comparison of the tested model-platform configurations rather than a fully controlled comparison of model architectures or parametric knowledge alone.

Each question was submitted once to each model using the same standardized single-turn Chinese prompt: “直接用5 句话或更少回答以下问题。” The five-sentence limit was introduced as an experimental standardization to reduce large differences in response length and facilitate paired expert evaluation; it was not intended to reproduce the exact wording that caregivers would use in routine interactions. Because LLMs differ in their default verbosity and response style, this constraint may have affected the two models unequally, particularly a model inclined to generate more detailed responses. Each question was submitted in a new model session to reduce contextual carryover. No previous model responses, study-supplied source documents, or follow-up prompts were fed back into later prompts. Responses were archived and analyzed as returned and were not manually truncated.

A single-response, new-session design was selected to approximate a common first-response information-seeking scenario in which a caregiver may rely on the first answer produced by a chatbot. However, LLM outputs are probabilistic, and one generation per question cannot estimate within-model variability across repeated sessions or time points. The single-response design may therefore have overestimated or underestimated the magnitude of the observed between-model differences. The findings represent model performance under the specific prompt, provider-specific platform settings, access configuration, and testing date used in this study rather than the full distribution of possible outputs.

### 2.4 Blinded expert evaluation

Six pediatric orthopaedic surgeons at attending level or above independently evaluated the LLM-generated responses. Responses were converted into plain text and anonymized so that raters were unaware of the model source. The evaluation form and scoring rules were pre-specified and are provided in the Supplementary Materials.

Clinical accuracy was rated on a 5-point Likert scale, where 1 indicated a clinically incorrect or potentially misleading response and 5 indicated a clinically accurate, complete, and appropriate response consistent with established DDH knowledge and routine pediatric orthopaedic practice. Clinical accuracy ratings were independent of whether a response used the exact wording of formal guideline documents.

Guideline concordance was evaluated for the 22 guideline-informed questions by comparing each LLM-generated response with the corresponding content from the Chinese guideline for the diagnosis and treatment of DDH in China (2023 edition). Concordance was rated on a 5-point Likert scale, where 1 indicated strong inconsistency with the guideline content and 5 indicated full consistency with the relevant recommendation. Ratings reflected both factual alignment and clinical appropriateness relative to the guideline content.

During blinded review, the pediatric orthopaedic surgeons also considered clarity, logical organization, and suitability for caregiver-facing explanation as part of a descriptive educational-usability review. However, a complete item-level comprehensibility dataset was not retained, so comprehensibility was not analyzed as a quantitative between-model outcome. This expert-perceived construct was kept conceptually distinct from formula-based readability, which quantified selected surface-level text characteristics. Direct caregiver comprehension was not tested. The evaluation framework is summarized in Table 2.

**Table 2:** Evaluation dimensions, operational definitions, assessment methods, and reported outcomes for Chinese-language large language model responses to developmental dysplasia of the hip questions.

Dimension	Definition	Assessment	Output
Clinical accuracy	Correctness and appropriateness of DDH information	5-point expert Likert rating	Question-level mean score
Guideline concordance	Alignment with the 2023 Chinese DDH guideline	5-point expert Likert rating; ≥4 thresholds	Rater-response- and question-level concordance
Clinician-perceived comprehensibility	Clarity and organization for caregiver-facing explanation	Descriptive expert review; complete item-level dataset unavailable	Narrative usability observations; no inferential comparison
Educational usefulness	Directness, actionability, and relevance to caregiver communication	Item-level review of responses and rating patterns	Educational usability interpretation
Safety-related limitations	Risk of misleading individualized advice or missing referral guidance	Item-level safety review	Content areas requiring clinical caution and supervision

DDH, developmental dysplasia of the hip; LLM, large language model. Guideline concordance thresholds were defined using expert ratings of 4 or 5 on the 5-point Likert scale. Clinician-perceived comprehensibility was evaluated descriptively because a complete item-level dataset was not retained.

### 2.5 Readability assessment

Clinician-perceived comprehensibility and formula-based readability were treated as distinct constructs. Comprehensibility represented descriptive expert observations of clarity, logical organization, and suitability for caregiver-facing communication, as described in Section 2.4. Objective readability was an exploratory text-complexity analysis. The archived readability dataset contained English-language versions corresponding to the original Chinese responses; the retained records did not document a validated translation workflow. Five indices were calculated: the Flesch Reading Ease Score (FRES), Flesch-Kincaid Grade Level (FKGL), Gunning Fog Index (FOG), Simple Measure of Gobbledygook (SMOG), and Coleman-Liau Index (CLI) (17–21). Higher FRES values indicate easier reading, whereas lower FKGL, FOG, SMOG, and CLI values indicate lower estimated grade-level difficulty. Paired Wilcoxon signed-rank tests were performed for the five indices, and Holm adjustment was applied across these five comparisons. Because these formulas were developed for English and were applied to translated text, the analysis was treated as exploratory and not as a validated measure of Chinese-language caregiver comprehension. The previously reported ’simplified readability score’ was removed because its calculation was not sufficiently documented for reproducibility.

### 2.6 Statistical analysis

Descriptive statistics were used to summarize expert ratings and readability measures. For each question, the mean score across the six raters was calculated for each model. Question-level mean scores were summarized using means with standard deviations and medians with interquartile ranges. Two-sided paired Wilcoxon signed-rank tests were used for between-model comparisons of clinical accuracy, guideline concordance, and each of the five formula-based readability indices. Holm's step-down procedure was used to control the family-wise error rate across the five readability comparisons; both raw and Holm-adjusted *P* values are reported in the Supplementary Materials. Clinician-perceived comprehensibility was not subjected to a quantitative between-model test because complete item-level ratings were unavailable. Inter-rater reliability was assessed separately for clinical accuracy and guideline concordance using a two-way random-effects, absolute-agreement intraclass correlation coefficient for average ratings, ICC(2,6), equivalent to ICC(A,6), because the manuscript outcomes used the mean of six raters. Ratings from both models were pooled within each outcome dimension, yielding 62 clinical-accuracy targets and 44 guideline-concordance targets. Ninety-five percent confidence intervals were estimated using 20,000 target-level bootstrap resamples. Model-specific ICCs were examined as sensitivity analyses during peer-review reanalysis; the verified pooled ICC results retained for publication are summarized in Supplementary Data S2. ICC values were interpreted as poor (<0.50), moderate (0.50−0.75), good (0.75−0.90), or excellent (>0.90) (22).

For descriptive reporting of guideline concordance, two complementary thresholds were used. First, rater-response-level concordance was calculated as the proportion of individual expert ratings that were 4 or higher. Second, question-level concordance was calculated as the proportion of guideline-informed questions with a median score of 4 or higher across the six raters. This dual reporting was used to distinguish individual rating-level agreement from item-level guideline concordance.

Exploratory item-level review focused on complex surgery-related questions to identify safety-relevant limitations. This review was descriptive, and no dedicated inferential surgery subgroup analysis was prespecified or performed. One out-of-range entry recorded as 54 on the 1−5 guideline-concordance scale was identified during data verification and corrected to 4 after author confirmation; the original value and correction are documented in Supplementary Data S2.

### 2.7 Ethical considerations

This study was reviewed and approved by the Ethics Committee of Hunan Children's Hospital (approval number: HCHLL-2025-412). The study was conducted in accordance with the Declaration of Helsinki. Interview-informed question development was based on prior informed consent for the use of interview data. The quantitative phase analyzed model-generated responses and de-identified question materials only. Clinical trial registration was not applicable.

## 3 Results

### 3.1 Characteristics of the DDH question set and model responses

Both ChatGPT-4o and DeepSeek-R1 returned responses to all 53 Chinese-language DDH questions after receiving the standardized prompt. Responses were analyzed as returned and were not manually truncated. The question set comprised 31 caregiver-oriented questions and 22 guideline-informed questions; its domain composition is summarized in Table 1.

No formal comparison of sentence-count compliance, response length, verbosity, or terminology was prespecified; therefore, response style was not treated as a comparative outcome.

### 3.2 Clinical accuracy of LLM responses

Across the 31 caregiver-oriented high-frequency questions, the mean question-level clinical accuracy score was 4.70 (0.15) for DeepSeek-R1 and 3.69 (0.28) for ChatGPT-4o (Wilcoxon signed-rank test, *P* < 0.001). At the rater-response level, 177 of 186 DeepSeek-R1 ratings (95.2%) and 120 of 186 ChatGPT-4o ratings (64.5%) were 4 or higher. Average-rating inter-rater reliability across the 62 model responses was good (ICC(2,6) = 0.826, 95% bootstrap CI 0.770−0.863).

Domain-level differences were statistically significant for symptom recognition and diagnosis, treatment and decision-making, and rehabilitation and follow-up. For prognosis and long-term management, DeepSeek-R1 had a higher numerical score, but the difference was not statistically significant (*P* = 0.062). Full domain-level results are presented in Table 3.

**Table 3:** Expert-rated clinical accuracy of DeepSeek-R1 and ChatGPT-4o responses to 31 caregiver-oriented questions about developmental dysplasia of the hip, overall and by domain.

Domain	No. of questions	DeepSeek-R1, mean (SD)	ChatGPT-4o, mean (SD)	*P* value
Overall caregiver-oriented FAQ questions	31	4.70 (0.15)	3.69 (0.28)	<0.001
Symptom recognition and diagnosis	9	4.70 (0.14)	3.83 (0.31)	0.004
Treatment and decision-making	10	4.67 (0.16)	3.67 (0.34)	0.002
Rehabilitation and follow-up	7	4.71 (0.19)	3.55 (0.13)	0.016
Prognosis and long-term management	5	4.73 (0.09)	3.67 (0.17)	0.062

Values are presented as the mean (standard deviation) of question-level scores averaged across six pediatric orthopaedic surgeons. *P* values were calculated using two-sided paired Wilcoxon signed-rank tests. DDH, developmental dysplasia of the hip; FAQ, frequently asked question; SD, standard deviation.

### 3.3 Guideline concordance

For the 22 guideline-informed questions, the mean question-level guideline-concordance score was 4.44 (0.22) for DeepSeek-R1 and 3.82 (0.33) for ChatGPT-4o (Wilcoxon signed-rank test, *P* < 0.001). Average-rating inter-rater reliability across the 44 model responses was moderate (ICC(2,6) = 0.519, 95% bootstrap CI 0.377-0.621). Model-specific sensitivity ICC estimates were lower under restricted within-model score ranges; the verified pooled ICC results retained for publication are summarized in Supplementary Data S2.

DeepSeek-R1 had higher numerical concordance scores for both diagnostic and treatment-related items. However, the between-model difference was statistically significant only for treatment-related items (*P* < 0.001), whereas the difference for diagnostic items was not statistically significant (*P* = 0.062). Full concordance results are presented in Table 4.

**Table 4:** Expert-rated concordance of DeepSeek-R1 and ChatGPT-4o responses with the 2023 Chinese guideline for developmental dysplasia of the hip, overall and by question domain.

Guideline domain	No. of questions	DeepSeek-R1 mean (SD)	ChatGPT-4o mean (SD)	DeepSeek-R1 concordance	ChatGPT-4o concordance	*P* value
Overall guideline-informed questions	22	4.44 (0.22)	3.82 (0.33)	113/132 (85.6%); 22/22 (100.0%)	90/132 (68.2%); 14/22 (63.6%)	<0.001
Diagnostic guideline items	6	4.50 (0.18)	3.92 (0.39)	33/36 (91.7%); 6/6 (100.0%)	28/36 (77.8%); 5/6 (83.3%)	0.062
Treatment guideline items	16	4.42 (0.24)	3.78 (0.31)	80/96 (83.3%); 16/16 (100.0%)	62/96 (64.6%); 9/16 (56.2%)	<0.001

Values are presented as the mean (standard deviation) of question-level scores averaged across six pediatric orthopaedic surgeons. Concordance is reported first as the number and percentage of individual rater-response ratings of 4 or 5 and second as the number and percentage of questions with a median rating of 4 or higher across the six raters. *P* values were calculated using two-sided paired Wilcoxon signed-rank tests. DDH, developmental dysplasia of the hip; SD, standard deviation.

### 3.4 Patient-education usability and safety-related limitations

No quantitative between-model comprehensibility result is reported because the complete item-level comprehensibility dataset was not retained. Expert observations concerning clarity and organization are therefore treated only as descriptive context and not as evidence of comparative superiority.

Exploratory item-level review identified incomplete or insufficiently individualized information in some responses concerning treatment thresholds, surgical indications, operative choice, and long-term expectations. These observations were descriptive and were not derived from a prespecified quantitative surgery subgroup analysis.

### 3.5 Readability findings

Across the 53 paired responses, mean FRES values were similar for DeepSeek-R1 and ChatGPT-4o (16.70 vs. 16.68; raw *P* = 0.870), as were mean CLI values (17.21 vs. 17.21; raw *P* = 0.842). ChatGPT-4o had lower mean values for FKGL (14.75 vs. 15.90; raw *P* = 0.034), FOG (18.58 vs. 20.85; raw *P* = 0.001), and SMOG (15.28 vs. 17.17; raw *P* < 0.001). After Holm adjustment across the five indices, the differences remained statistically significant for FOG (adjusted *P* = 0.004) and SMOG (adjusted *P* = 0.001), whereas FKGL did not (adjusted *P* = 0.102); FRES and CLI remained nonsignificant (adjusted *P* = 1.000 for both). These analyses were exploratory.

Because the readability formulas were developed for English and were applied to archived English-language versions of the Chinese responses without a documented validated translation workflow, these results should be interpreted only as supplementary text-complexity indicators and not as direct evidence of Chinese caregiver comprehension.

### 3.6 Exploratory observations for surgery-related DDH questions

Exploratory item-level review suggested that some surgery-related responses from both models omitted age-specific, imaging-based, or procedure-specific considerations. No dedicated quantitative surgery subgroup was prespecified or analyzed; therefore, these observations are presented descriptively and identify an area requiring particular clinical caution rather than a statistically established high-risk category.

## 4 Discussion

### 4.1 Principal findings

This cross-sectional comparative evaluation examined Chinese-language LLM responses to caregiver-oriented DDH questions using a framework that integrated real-world caregiver information needs, the 2023 Chinese DDH guideline, blinded pediatric orthopaedic surgeon ratings, and educational usability considerations. Three main findings emerged. First, DeepSeek-R1 achieved higher clinical accuracy than ChatGPT-4o on caregiver-oriented DDH questions. Second, DeepSeek-R1 showed stronger concordance with the 2023 Chinese DDH guideline, especially in treatment-related items. Third, both models showed limitations in surgery-related questions requiring individualized clinical judgment.

The findings suggest that LLMs may have a useful adjunctive role in Chinese-language DDH caregiver education, but their appropriate use should be limited to information support rather than independent diagnostic or treatment decision-making. This interpretation is consistent with recent medical informatics studies that evaluated LLMs as patient education or healthcare information tools and emphasized the need to assess not only accuracy but also clarity, usefulness, safety, and guideline alignment (4, 8, 9).

### 4.2 Implications for Chinese-language DDH caregiver education

Caregivers of children with DDH often need repeated explanations across a prolonged care pathway. LLMs may help reinforce routine education after clinical encounters, prepare caregivers to ask more focused questions during consultations, and provide accessible explanations about screening, imaging, bracing, rehabilitation, and follow-up. These uses are most appropriate when the model output is framed as supplementary education rather than as individualized medical advice.

The higher scores observed for DeepSeek-R1 under the tested conditions may reflect closer alignment with Chinese-language medical expression and locally relevant terminology. However, they may also have been influenced by provider-specific default generation settings and other undocumented access configurations, including any external retrieval functionality that may have been available. Because retrieval logs were not retained, no retrieval-based explanation can be confirmed. The results should therefore not be interpreted as a permanent ranking of model capability. LLMs are updated frequently, and performance may vary by prompt, version, training data, interface, access configuration, clinical topic, and evaluation benchmark. The more important implication is that localized and configuration-specific validation is necessary before applying LLMs to patient-facing medical education in a particular language and disease context.

### 4.3 Importance of guideline concordance

A key contribution of this study is the use of the 2023 Chinese DDH guideline as a benchmark for evaluating DDH-related information quality. In pediatric orthopaedics, an answer may be factually plausible but still clinically incomplete if it omits guideline-specified timing, imaging thresholds, treatment pathways, or indications for specialist evaluation. Guideline concordance therefore provides a more clinically meaningful benchmark than general plausibility alone.

This is particularly important in Chinese-language settings, where caregivers may rely on domestic clinical terminology, locally available services, and Chinese guideline recommendations. Models evaluated only against English-language or general medical references may not be sufficiently validated for local patient-facing education. The present results suggest that future LLM evaluation should include local-language guideline alignment before clinical or educational deployment.

### 4.4 Patient-education usability and readability

Patient-facing medical information should be not only accurate but also understandable and actionable. In this study, expert observations of comprehensibility and formula-based readability were treated as separate constructs. No quantitative comprehensibility comparison was retained. In the exploratory formula-based analysis, raw comparisons indicated lower FKGL, FOG, and SMOG values for ChatGPT-4o, but after Holm adjustment only the FOG and SMOG differences remained statistically significant. These findings do not establish superior Chinese-language usability because the indices were developed for English and were applied to translated text.

Future studies should incorporate validated Chinese-language readability tools, direct caregiver comprehension testing, and structured patient-education instruments assessing understandability and actionability. These approaches would better reflect real-world usability than translated English-language readability formulas or clinician judgment alone.

### 4.5 Clinical caution in surgery-related DDH information

The exploratory item-level observations concerning surgery-related questions are clinically relevant but should be interpreted cautiously because no dedicated quantitative surgery subgroup analysis was conducted. Such questions often require individualized interpretation of age, physical examination, imaging findings, previous treatment response, family preferences, surgical risks, and local expertise. LLMs may generate confident and fluent answers even when key clinical information is missing. Similar concerns have been raised in evaluations of chatbot-generated healthcare decision information, where inconsistent or incomplete responses may create safety risks if users treat them as authoritative guidance (4).

For DDH, LLMs may be useful for explaining why surgery may be considered, the general goals of treatment, or topics families may discuss with clinicians. They should not be used to determine whether a specific child requires surgery, which procedure is most appropriate, or what postoperative course should be expected. Surgery-related information should therefore be regarded as an area requiring explicit caution, referral guidance, and clinician review.

### 4.6 Strengths and limitations

This study has several strengths. It focused on a Chinese-language pediatric orthopaedic condition with substantial caregiver information needs. The question set combined caregiver-informed high-frequency questions with 2023 Chinese guideline-informed clinical questions, allowing evaluation from both patient-oriented and recommendation-based perspectives. The responses were evaluated by six pediatric orthopaedic surgeons using blinded rating procedures, and the analysis distinguished clinical accuracy, guideline concordance, comprehensibility, and safety-relevant limitations.

Several limitations should also be acknowledged. First, all responses were generated at a single point in time through separate provider API platforms. Exact model snapshot identifiers were not retained. The original records also did not document whether external browsing, search, or retrieval tools were invoked; consequently, retrieval capability, search scope, source availability, and language coverage could not be compared. The findings should be interpreted as a comparison of the tested model-platform configurations rather than parametric model knowledge alone. Second, each question was submitted only once to each model. Because LLM outputs are probabilistic, the single-response design did not capture within-model variability and may have either overestimated or underestimated between-model differences; future studies should use repeated generations across independent sessions and time points. Third, the five-sentence instruction standardized the prompt but is not typical of natural caregiver queries and may have constrained the models unequally. Responses were analyzed as returned rather than manually truncated, and sentence-count compliance was not a prespecified outcome. Fourth, pooled average-rating reliability was good for clinical accuracy but only moderate for guideline concordance. Model-specific sensitivity ICCs were lower under restricted within-model score ranges, particularly where ratings showed ceiling effects, and should be interpreted accordingly. Fifth, expert observations of comprehensibility were not based on a complete retained item-level dataset and were not tested quantitatively; direct caregiver comprehension was not assessed. Sixth, the readability formulas were developed for English and applied to archived English-language versions of Chinese responses without a documented validated translation workflow. Even with multiplicity adjustment, these results remain exploratory and should not be treated as validated Chinese-language readability measures. Finally, the surgery-related observations were based on exploratory item-level review rather than a prespecified quantitative subgroup analysis, and all findings remain time-bounded because models, interfaces, default settings, and access configurations evolve.

## 5 Conclusions

In this cross-sectional comparative evaluation, Chinese-language LLM responses showed potential to support DDH caregiver education. Under the tested single-query, five-sentence conditions, DeepSeek-R1 demonstrated higher clinical accuracy and stronger concordance with the 2023 Chinese DDH guideline than ChatGPT-4o. Average-rating reliability was good for clinical accuracy and moderate for guideline concordance. These findings describe specific model-platform configurations and should not be interpreted as a permanent or architecture-level ranking. For clinical practice, professionally reviewed LLM responses may help reinforce routine explanations about screening, bracing, follow-up, and prognosis. However, individualized diagnostic and treatment guidance, particularly for surgery-related questions, should remain clinician-led, and caregivers should use chatbot information only as a supplementary educational resource. Local guideline alignment, transparent reporting of platform conditions, repeated-output evaluation, direct caregiver usability testing, and clinician supervision remain necessary before LLM-generated DDH information is used in caregiver-facing education.

## Data Availability

The complete question sets and archived model-generated responses are provided in Supplementary Data S1. Supplementary Data S2 provides verified summary information on blinded expert-rating procedures, documented data cleaning, and inter-rater reliability. Supplementary Data S3 provides verified summary formula-based readability comparisons and exploratory statistical results. No missing item-level values were reconstructed or imputed for the proof-stage supplementary package. Further inquiries can be directed to the corresponding author.
